# Food Traceability: A Consumer-Centric Supply Chain Approach on Sustainable Tomato

**DOI:** 10.3390/foods10030543

**Published:** 2021-03-05

**Authors:** Foivos Anastasiadis, Ioanna Apostolidou, Anastasios Michailidis

**Affiliations:** Deptartment of Agricultural Economics, School of Agriculture, Aristotle University of Thessaloniki, 541-24 Thessaloniki, Greece; ioanapost3@yahoo.gr (I.A.); tassosm@auth.gr (A.M.)

**Keywords:** food supply chains, food safety, tomato, Greece, end-to-end approach, sustainability

## Abstract

Technological advances result in new traceability configurations that, however, cannot always secure transparency and food safety. Even in cases where a system guarantees transparency, the actual consumer involvement and a real consumer-based perspective cannot always be ensured. The importance of such consumer centricity is vital, since it is strongly associated with effective supply chains that properly fulfil their end-users’ needs and requests. Thus, the objective of this paper was to explore the level of consumer centricity in food supply chains under a traceability system. The methodological approach employed a framework of two studies validating subsequently a similar set of variables, using initially consumers data and then supply chain actors data. The supply chain of sustainable tomato was selected to design the studies. The level of agreement between datasets suggested the level of the supply chain consumer centricity. Findings showed health, trust, quality, nutrition, and safety-related values to be significant for the consumers towards accepting a traceability system. The supply chain actors also accepted a traceability system based on the fact that their customers’ needs rely on the exact same beliefs, indicating a high level of consumer centricity. The current work underlines the magnitude of consumer centricity in food supply chains and provides an easy and straightforward framework for its exploration. Key implications suggest the design of more effective supply chain and consumer-based strategies for the food industry. Policymakers could also adopt the concept of consumer centricity to further improve the food industry.

## 1. Introduction

Consumers by default are a vital part of any supply chain (SC). According to several supply chain definitions [1,2] and, most importantly, based on the supply chain management concept [3,4], the entire supply chain should serve the end-user of the product/service produced and fulfilled by the supply chain, i.e., the consumer. Nonetheless, theoretical and academic approaches usually are quite far from reality. The continuous pressure on the economic viability of businesses—and eventually their supply chains—results in an overall focus on high revenues and even higher margins [5]. Consequently, in practice (versus theory), there are quite a few cases were supply chains choose to operate against their end-users needs. This could be due to the absence of cooperation among different supply chain actors, challenging the integration of key supply chain processes, and to a myopic view focused on specific levels rather than on the entire chain [6]. The repeated food scandals in food supply chains (for instance, horse meat [7], eggs–fipronil [8], pork–hepatitis [9]) perfectly illustrate the above point.

Nowadays, consumers pay close attention to food safety, requesting high-quality food with information transparency and safety assurances [10]. Food traceability systems can reduce information asymmetry, implement safety governance across geographical boundaries, and help increase consumer faith in food safety to achieve a sustainable agricultural development [11]. From a consumer perspective, traceability helps to build trust, provide peace of mind, and increase confidence in the food system. For the growers, traceability is part of an overall cost-effective quality management system that can also assist in continuous improvement and minimization of the impact of safety hazards. It also facilitates the rapid and effective recall of products and the determination and settlement of liabilities [12]. The implementation and benefits of a traceability system depend on consumers’ awareness of food safety, thus an increasing need for such systems that provide transparent information on the quality and safety of food supply chains. The greatest benefit of food traceability could be realized when it is implemented at the entire supply chain level and engages all the participants involved [13]. Therefore, there is a conceptual link between food traceability and consumer-centric supply chains.

The current study aims to further explore the level of consumer centricity in food supply chains implementing traceability systems. Such an investigation shall reveal any association between these concepts. Greek sustainable tomato supply chain was chosen as the research area, due to the high production and consumption of tomaotes in Greece. Two different surveys took place, one based on the consumers and the other on the supply chain actors. The analysis resulted in tangible evidence regarding the relationship between traceability and consumer centricity. The analysis also provides further insights into the nature and importance of consumer-centric food supply chains.

## 2. Materials and Methods

In this section, initially, we separately present the reviews of key studies on consumer-centric food supply chains and food traceability. Afterwards, the relationship between these concepts is further explored in the literature, revealing a specific research gap. Finally, we describe the methodology employed.

### 2.1. Consumer-Centric Supply Chains

Every company claims a customer-driven strategy. However, the diverse interpretation of their customers’ needs by the numerous value-chain entities, either external, i.e., suppliers and retailers, or internal like R&D managers, results in loss of focus. Thus, a need for frameworks and further research that can help companies develop strategies that are actually customer-centric [14]. This sub-section presents and discusses several examples of consumer-centric approaches in food supply chains. These cases employ diverse methods, have different specific objectives, and use various terminologies (e.g., consumer-driven, customer-oriented, demand-driven, consumer co-creation in new product development), yet all are relevant to the same concept: consumer-centric food supply chains.

A study on the organic tomato supply chain in The Netherlands recommends shifting from the Supply Chain Management concept to Value Chain Management and ultimately to move towards more customer-based strategies. It suggests considering consumers’ sustainability concerns in the direction of “greening” the supply chains end to end, gaining competitive advantage through a better position of the entire firm’s “greening” [15]. Another study on the blueberry supply chain in Italy highlights the impact of a demand-driven supply chain towards the activation of innovation processes throughout the fresh fruit supply chains. This case exemplifies the integration between a business firm and a research centre, resulting in a new consumer-centred dialogue with the market. The main output was a novel packaging system enabling the maintenance of the quality of the fruits for a longer period, improving the exports, increasing the turnover of the associated group, and improving the remuneration of the fruit growers [16].

Research on pork supply chains towards delivering superior eating quality to consumers explored why there is more emphasis on mass production—which usually is combined with traceability systems and high food safety procedures—than on essential quality cues. A more consumer-centric approach suggests combining a hedonic assessment of meat attributes end to end in the value chain, revealing the complexity regarding to the formation, communication, and provision of attribute-related information to the final consumers [17]. Product modularity and customization to consumer’s requirements is another form of consumer-centric supply chain approach. A study investigating the relationships between sustainability, operations, and marketing suggests that such configurations may generate higher demand due to greater satisfaction provision. They also reveal an association between modularity and the development of sustainable products, which, in turn, may enhance sustainable consumption [18].

New product development and co-creation with the active involvement of the consumers is a key element in the consumer-centric supply chain configuration. A paper exemplifies a novel methodology for the creation and design of new beverages, employing a digital crowdsourcing tool to reveal consumer preferences. Afterwards, these preferences were transformed into actionable directions, that allowed a rapid production of the beverage for tasting and evaluation. Hence, a closed-loop beverage design method based on consumers co-creation was applied [19]. Another new product development framework highlighting the importance of consumer-centric supply chains suggests the development, integration, and triangulation of personalized food profiles for customized healthier products [20]. Such reconfigurations suggest digital manufacturing and modularity principles for securing an authentic production–consumption alignment while producing personalized healthier food products [6].

### 2.2. Food Traceability

The food industry is becoming more customer-oriented and needs faster response times to deal with food scandals and incidents. An effective traceability system helps to minimize the production and distribution of unsafe or poor-quality products, thereby minimizing the potential for bad publicity, liability, and recalls. The current food labelling system cannot guarantee that the food is authentic, safe, and of good quality. Therefore, traceability is applied as a tool to assist in the assurance of food safety and quality as well as to achieve consumer confidence [10]. Food traceability has gained considerable importance, particularly following several food safety incidents during which traceability systems were weak or absent [21]. Traceability appears as a tool to comply with legislation and to meet food safety and quality requirements. It is considered to be an effective safety- and quality-monitoring system with the potential to improve safety within food chains, as well as to increase consumer confidence and to connect producers and consumers [11].

To supply top-quality, safe, and nutritious foods, as well as rebuild public confidence in the food chain, the design and implementation of whole chain traceability from farm to end-user have become an important part of the overall food quality assurance system [12]. FAO stated that managing food safety and quality should be a shared responsibility of all actors in the food chain including governments, industry and consumers [22]. Because of globalization in the food trade, effective food control systems are essential to protect the health and safety of consumers. The foremost responsibility of food control is to enforce the food law(s) protecting the consumer against unsafe, impure, and fraudulently presented food [23]. Food traceability systems can provide a reliable and continuous information flow in supply chains, identify root causes of problems, and recall high-risk products from the market [24].

Therefore, food traceability systems can reduce consumer information asymmetry and food safety risks [25,26,27]. Given that traceability is mainly a quality assurance tool, its implementation depends on many factors linked to the supply chain [28,29] and trade-related issues [30]. The use of traceability as a tool to increase consumer confidence in food safety is also mainly linked to distrust in the food system that should be addressed by the government [30,31,32]. 

### 2.3. Research Gap

The increasing consumer interest on short food supply chains highlights the importance of more information and more direct contact with the producers [33], revealing the need for innovative traceability systems and further active consumer involvement in the food supply system; hence, more attnetion on consumer-centric supply chains. Recent technological advances have significantly contributed to traceability in food supply chains. For instance, blockchain technology increases trust, authenticity, and transparency [34]. However, there are several challenges such as the need for Internet-of-Things devices throughout the food supply chain to capture all the necessary data from several parties (supply chain actors) and transfer the data to the blockchain [35]. Moreover, blockchain technology is quite new, and long-term impacts of its implementation in the food chain are not yet proven [34]. Besides, with this technology, there is no actual consumer involvement, thus no consumer centricity in the supply chain.

The paradigm of blockchain technology reveals an issue regarding the role of traceability and transparency per se in the food supply chains. The approaches about consumer-oriented traceability are limited in the literature (as illustrated above in Section 2.1 and Section 2.2). However, some studies highlighted the fact that consumers value a bi-directional connection to the supply chain through the ability to give feedback via a consumer-oriented food traceability app, supporting in this way sustainable food consumption [36]. In the same direction, another study revealed the importance of customer-delivered value of a food traceability system, that contributes to enhancing their intention to purchase traceable food [37]. Despite the above examples, the majority of extant research on food traceability has taken either a firm-centric approach or a reflexive and non-thorough consumer-based orientation. Whether and how food supply chain actors value their consumers’ motivation for a traceability system—including their relevant needs and requests concerning such system—remain to be explored in significant detail. It seems appropriate and necessary, then, to unpack the food supply chain consumer centricity concept for the case of traceability.

### 2.4. Methodology

The review of previous studies on consumer-centric food supply chains and food traceability revealed a gap in the literature concerning the way the entire supply chain responds to consumers’ claims and requests. The case of traceability is the ideal scenario to test the level of consumer centricity in food supply chains due to the, by default, involvement of all the stakeholders through the entire supply chain. Given the certain contribution of the retailers, wholesalers, logistics, manufactures, and producers at the respective supply chain level, a traceability system requires strong collaboration among all its participants to properly function. The question, however, is whether the supply chain actors collaborate under their personal agendas and respond to their customers’ needs and requests. The literature review shows a strong possibility of “accidental” response to consumers requests, e.g., no consumer involvement (ergo, no consumer-centricity), yet a satisfactory reaction to transparency claims. Thus, the objective of the study is to explore in depth several aspects of consumer centricity to conclude whether its level is satisfactory throughout the supply chain. An overall research question of the current study could be formulated as follows:

What is the level of consumer centricity in food supply chains applying a traceability system?

**Figure 1:**
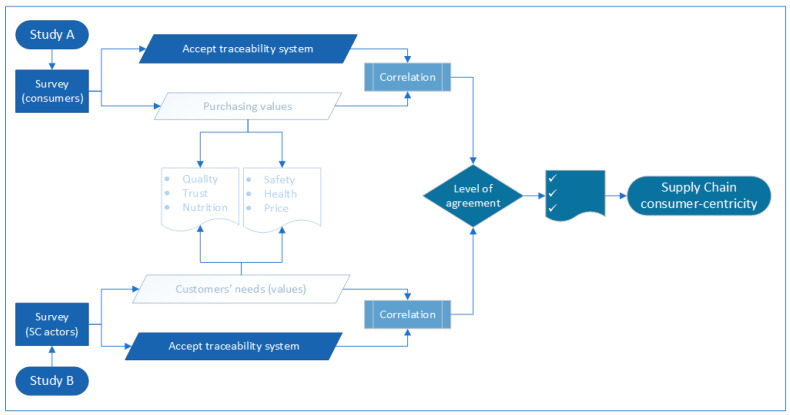
Methodological framework (source: authors).

To explore such a research question, we designed two studies. The first study (study A) is a consumer survey to identify consumers’ perceptions and concerns regarding a traceability system. Similarly, the second study (study B) is a survey on supply chain stakeholders (i.e., retailers, wholesalers, manufactures, and producers), exploring whether they accept a traceability system based on their consumers’ perceptions for such a system. As illustrated in the methodological framework (Figure 1), the level of agreement between consumers and supply chain actors on the significant values associated with a traceability system indicates the supply chain’s level of consumer centricity.

The supply chain of sustainable tomato was selected to design the studies. That includes all supply actors from the production to the consumption of the tomatoes produced, processed, and sold with sustainable practices, for example, under organic or integrated management. This is essential, so we can present a consistent scenario, with a specific product, to the consumers during the survey in study A and more importantly to target all the stakeholders through the entire supply chain in study B. Regarding data collection, we selected the Metropolitan area of Thessaloniki in Greece as a research area for both studies, due to the high consumption of tomatoes and the availability in the region of the necessary supply chain stakeholders [38].

For study A, we designed and disseminated a questionnaire using SurveyMonkey, exploring consumers’ acceptance of a traceability system for sustainable tomato. In addition, we asked consumers to rank the importance of certain purchasing values focused on issues related to: (i) health, (ii) quality, (iii) price, (iv) trust, (v) nutritional value, and (vi) food safety. Most of these questions were framed in five-point Likert-scale intervals, so as to encourage participation and minimize the cognitive burden on the respondents. As a result, the key factors revealed were Quality, Trust, Nutrition, Health, Safety, and Price, consistently with previous studies [39,40,41,42,43]. The sampling method involved an online random sample [44]. The questionnaire was circulated for 4 months and resulted in 729 valid responses.

For study B, we also stuctures and circulated a similar questionnaire using SurveyMonkey, exploring supply chain stakeholders’ acceptance of a traceability system for sustainable tomato. Moreover, the participants had to rank the importance of their customers’ specific values (using a five-point Likert scale) about their decision to accept a traceability system. The key values used were the same six values from study A, resulting in the same factors (Quality, Trust, Nutrition, Health, Safety, and Price) plus three more business-oriented values: Reliability, Better Market Access, and Stakeholders’ Sustainability [45,46]. In this study, the sample was selected following the expert sampling technique [47]. Participants were experts in the area of study, and this sampling method also involved sample assembling of a group of people that could use their experience. The reason for using expert sampling was to have a better way of constructing the views of individuals that were expert in a definite area. The sampling objective was to include representatives from the entire tomato supply chain, i.e., producers, middlemen, wholesalers, manufactures, logistics, and retailers. Given the significantly smaller number of supply chain stakeholders compared to consumers and the objective to achieve—an analogy among the available stakeholders in every category and those in our sample—we concluded with a sample of 69 valid responses.

## 3. Results

Initially, in this section, we separately present, in their respective subsections, the analysis and the results of the two studies. Then, we discuss these results by reflecting on the methodological framework and the status of the supply chain consumer centricity.

### 3.1. Study A (Consumers)

Descriptive statistics of the demographic characteristics (Table 1) suggested a representative sample based on other related studies [48,49,50]. Reliability analysis confirmed that the scale was reliable, Cronbach’s alpha coefficients ranged from 0.712 to 0.809, exceeding the minimum standard of 0.60 suggested by Malhotra [51].

Bivariate Spearman correlation was employed, since Pearson’s correlation assumptions were violated [52], to explore the relation between Accept traceability system and the six purchasing factors (Quality, Trust, Nutrition, Health, Safety, and Price). Table 2 presents the output of the statistical analysis using SPSS. Quality, Trust, Nutrition, Safety, and Health were positively related to Accept traceability system, with a correlation coefficients rho = 0.129, rho = 0.144, rho = 0.156, rho = 0.218, and rho = 0.156, respectively, which were also significant at *p* < 0.01 probability. Only Price was not statistically significantly related to Accept traceability system.

### 3.2. Study B (SC Stakeholders)

The sampling procedure (as justified above in Section 3) suggested a representative sample, including a sufficient number of every stakeholder through the entire supply chain. Reliability analysis confirmed that the scale was reliable, with Cronbach’s alpha coefficients rangein from 0.805 to 0.913, exceeding the minimum standard of 0.60 suggested by Malhotra [51].

Bivariate Spearman correlation was employed, since Pearson’s correlation assumptions were violated [52], to explore the relation between *Accept traceability system* and the six customers’ values (Quality, Trust, Nutrition, Health, Safety, and Price) plus the three business-related variables (Reliability, Better Market Access, and Stakeholders’ Sustainability). Table 3 presents the output of the statistical analysis using SPSS. Trust, Health, Safety, Quality, Nutrition, Price, and Reliability awee positively related to Accept traceability system, with correlation coefficients rho = 0.612, rho = 0.539, rho = 0.584, rho = 0.422, rho = 0.564, rho = 0.341, rho = 0.442, respectively, which were also significant at *p* < 0.01 probability. Stakeholders’ Sustainability, and Better Market Access were positively related to Accept traceability system, with correlation coefficients of rho = 0.277 and rho = 0.343, significant at *p* < 0.05 and *p* < 0.01, respectively.

## 4. Discussion

This section discusses the results, reflecting on the supply chain consumer centricity dimention. Study A resulted in five (out of six) significant factors related to the acceptance of a traceability system. That means consumers have a positive perception of a traceability system. Specifically, applying such a system to the sustainable tomato supply chain enhances consumers’ trust in the entire supply chain. Also, it improves their perception of a product’s quality and its nutritional value, while increasing their sense of product’s safety. Overall, applying a traceability system to food products makes consumers believe that these products are more beneficial to their health. These findings are consistent with previous studies [24,53].

Study B resulted in seven (out of nine) significant factors related to the acceptance of a traceability system. That means sustainable tomato supply chain actors have a positive perception about a traceability system. Particularly, the supply chain stakeholders believe that the acceptance of a traceability system would increase their customers’ trust. Moreover, they believe that it would improve their customers’ perception of the nutritional value and both the safety and the quality of their products. Overall, they believe that such a system makes their customers believe that traceable products protect their health. On top of these customer-related values, supply chain stakeholders believe that a traceability system will have a positive effect on their reliability and on products’ prices—thus strengthening their reputation—which is consistent with their previous positive perceptions. Past studies also suggest a similar supply chain stakeholder’s behavior [54], though not necessarily related to their customers’ perceptions.

Comparing the output of both studies, according to the methodological framework, we should highlight the absolute matching among the significant purchasing values of the consumers and the supply chain actors’ perceptions on their customers’ needs and requests (values). Reviewing such an outcome from a supply chain perspective results in prominent conclusions concerning the consumer centricity of a sustainable tomato sector that has adopted a traceability system. The tomato supply chain end-users (the consumers) appreciate the positive effect of a traceability system on the product’s quality, safety, and nutritional value, on their trust for these products, and overall, on their health. Identically, the rest of the supply chain actors (the retailers, wholesalers, logistic operators, manufactures, and producers) value in a similar manner the exact same beliefs, considering the positive effect of a traceability system on their customers. In other words, the supply chain responds to the requests of the consumers and considers adopting a traceability system following a uniform end-to-end logic. This a priori test of traceability-related variables among consumers and subsequently multiple supply chain actors is the main conceptual contribution of the current study.

The set of variables (purchasing-related values) tested in study A and next tested in study B (as customers’ needs according to supply chain actors) corresponds to the depth of a consumer centricity exploration: the larger the set of variables, the more thorough the exploration. Similarly, the higher the agreement on these variables among consumers and supply chain stakeholders, the higher the level of consumer centricity. This means that adopting a traceability system for such a supply chain shall be very well appreciated by its consumers.

## 5. Conclusions

Findings from study A suggested consumer’s acceptance of a traceability system for a sustainable tomato supply chain based on the significance of quality, trust, nutrition, safety, and health-related values. Similarly, study B verified that the supply chain actors accept a traceability system based on the fact that their customers’ needs rely on the exact same beliefs, indicating a high level of consumer centricity. Reaching such a conclusion before investing the implementation of a food traceability system is extremely valuable. It is not just a positive signal regarding the supply chain effectiveness but also predefines a significant consumer engagement in the presence of a traceability system. In line with previous studies [36], such level of supply chain consumer centricity indicates a bi-directional connection that could result in interactive communication between consumers and multiple supply chain actors.

Underling the magnitude of supply chains’ consumer centricity is the key contribution of the current study. The framework employed to achieve this is also significant. Despite the availability of a few studies on consumer-oriented supply chains [14,16,17,55,56], the extant literature fails to provide a straightforward approach to explore the actual consumer centricity at a supply chain level.

The obvious implication of the current research findings is a recommendation to apply the framework to the existing traceability systems and further improve these systems by adopting consumer involvement and engagement principles. Moreover, there are wider applications that go beyond just a positive impact on traceability systems, with several managerial and policy-making implications. Concerning the former, a consumer centricity assessment shall be part of any management toolbox, especially among C-level executives and decision-makers. A firm’s strategy and the design of future investments must be viewed from a consumer’s perspective. Regarding the latter, the food industry, and generally, the agricultural sector could also benefit from shifting their policy towards a more consumer-centric direction. Policymakers should realize that the entire food system has to work towards fulfilling consumers’ needs. Therefore, the current study establishes a necessity of better understanding the importance of supply chains consumer centricity and adopting its principles in the policy-making process.

Limitations of the study and, simultaneously, recommendations for further research regard the generalization of the findings due to the fact that this work was based on a single case and a national sampling. Similar studies should be designed in several countries for multiple food supply chains, covering many types of products, i.e., vegetables, meat, and dairy. Moreover, additional variables and multivariate statistical analyses could enhance the current framework. Nevertheless, caution is necessary about adding variables, which would increase the complexity of the system and its analysis. In fact, one of the objectives (and also key advantages) of the framework is the fact that it is easy and straightforward.

## Figures and Tables

**Table 1:** Descriptive statistics of consumers’ demographic characteristics.

Education			
		Frequency	Percent
Valid	High school	71	9.7
	University	329	45.1
	Masters	178	24.4
	PhD	153	20.7
	Total	729	100.0
Gender			
Valid	Female	474	65.0
	Male	255	35.0
	Total	729	100.0
Age			
Valid	18–24	135	18.5
	25–34	138	18.9
	35–44	173	23.7
	45–54	164	22.5
	55–64	96	13.2
	65+	23	3.2
	Total	729	100.0
Annual Household Income			
Valid	<€7999	126	17.3
	€8000–€14,999	202	27.7
	€15,000–€24,999	190	26.1
	€25,000–€39,000	154	21.1
	€40,000–€59,999	44	6.0
	€60,000–€74,999	7	1.0
	€75,999–€99,999	6	8
	Total	729	100.0
Family Status			
Valid	Married	354	48.6
	Single	375	51.4
	Total	729	100.0

**Table 2:** Correlations (consumers).

Accept Traceability System	Spearman Correlation Coefficient (*rho*)
Quality	0.129 **
Trust	0.144 **
Nutrition	0.156 **
Safety	0.218 **
Heath	0.156 **
Price	0.029

** Indicates that correlation is significant at the 0.01 level (two-tailed).

**Table 3:** Correlations, supply chain (SC) stakeholders.

Accept Traceability System	Spearman Correlation Coefficient (rho)
Increase consumers’ trust	0.612 **
Protect consumers’ health	0.539 **
Increase food safety	0.584 **
Better food quality	0.422 **
Positive on products nutritional value	0.564 **
Improve stakeholders’ sustainability	0.277 *
Product price	0.341 **
Stakeholders’ reliability	0.442 **
Better market access	0.343 **

*, ** Indicates correlation is significant at the 0.05 level (two-tailed) and 0.01 level (two-tailed), respectively.

## Data Availability

Not applicable.
